# Changes in sequencing technology used for preimplantation genetic testing for aneuploidy

**DOI:** 10.1007/s10815-025-03576-5

**Published:** 2025-07-11

**Authors:** Vivianne Elizabeth Oltramare, Catherine McDermott, Paul Joseph Dunn

**Affiliations:** Faculty of Health Sciences & Medicine, Bond University, 14 University Drive, Robina QLD 4226, Gold Coast, Australia

**Keywords:** PGT-A (preimplantation genetic testing for aneuploidy), Nanopore, NGS (next generation sequencing), Microarray, FISH (fluorescence in situ hybridisation)

## Abstract

The continued development of assisted reproductive technologies has aimed to improve pregnancy outcomes through screening methods for the identification of embryos with monogenic disorders, chromosomal aneuploidy or structural abnormalities. There is an increased risk of chromosomal abnormalities for patients with advanced maternal age, recurrent implantation failure and recurrent pregnancy loss. To address this, preimplantation genetic testing for aneuploidy has evolved through several methods since the 1990s, when it first began through fluorescence in situ hybridisation. The limitations of these early methods were overcome with the progression in technology that enabled the screening of all 23 chromosome pairs. These methods included microarray methods and next-generation sequencing platforms. Currently, these are used for PGT-A; however, for IVF clinics to carry out PGT-A utilising these methods, samples are sent to external laboratories capable of carrying out these methods. More recently, nanopore sequencing has emerged, which may overcome these limitations and be carried out within IVF clinics to facilitate faster patient results. This review focuses on the evolution of PGT-A methods through next-generation sequencing and the current status of these methods.

## Background

Approximately 10–15% of all first-trimester pregnancies will result in pregnancy loss [1]. Recurrent miscarriages, classified as three or more consecutive pregnancy losses, affect 1–2% of all couples, the risk increases with age going from 0.13% for women under 25 to 13% for women over 40 [1–3]. Factors that contribute to miscarriage include the following: endocrine factors (diabetes, polycystic ovarian syndrome); environmental agents (alcohol, smoking) and chromosomal and single gene disorders (foetal chromosomal abnormalities, parental balanced translocations, thrombophilia and x-linked male conditions) [4]. Advanced maternal age is also known to have a substantial effect on spontaneous abortions, specifically as age plays a large part in increased chances of chromosomal abnormalities [3, 5].

Chromosomal abnormalities can occur in many different forms and are a leading cause of miscarriage and spontaneous abortions, especially in the first trimester of pregnancy, where such abnormalities are responsible for 50% of these losses [1, 6]. A gain or loss of chromosomal material caused by numerical chromosomal abnormalities can lead to an over or under-expression of genes that result in pregnancy loss [7]. These can be related to gains (trisomy) or losses (monosomy) of single chromosomes, broadly grouped as aneuploidy, or through gains and losses of entire sets of chromosomes (polyploidy), such as triploid (three sets of chromosomes) or tetraploid (four sets of chromosomes). This gene dosage alteration affects the genes vital for embryo development, leading to loss of pregnancy, implantation failure, stillbirths, neonatal deaths, intellectual and developmental disabilities or identifiable disorders in an affected baby [8, 9].

**Fig. 1:**
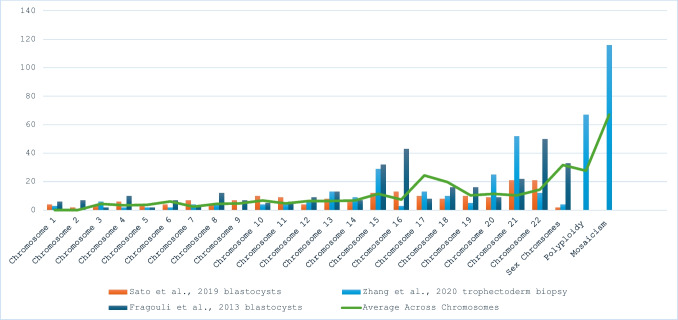
Comparison of the distribution of chromosomal abnormalities across three papers. Three papers included the distribution of abnormalities across chromosomes and further abnormalities. Each study used various methods, from trophectoderm biopsy of blastocysts for couples undergoing IVF with PGT-A [10–12]. Methods used aCGH and SNP arrays. Zhang et al. looked at 220 PGS cycles which included couples that underwent fertility assessment before embryo transfer; Sato et al. included 83 patients with recurrent pregnancy loss from the ages of 35–42; Fragouli et al. included 118 couples undergoing fertility treatment through IVF [10–12]

The frequency and distribution of chromosomal abnormalities across all chromosomes within embryos that miscarried or resulted in spontaneous abortion are usually random; however, some chromosomes are more susceptible to abnormalities compared to others (Fig. 1) [10–12]. Aneuploidy is most commonly found in chromosomes 15, 16, 21 and 22 [10–12]. Even though aneuploidies of chromosomes 16 and 22 are common, they are lethal in all cases due to the high concentration of genes found on the chromosomes [1]. Therefore, identifying such abnormalities is vital to determine the best course of treatment and prevention. This narrative review will cover the changes in PGT-A methods and the importance of the advances from the early 1990 s to the present day made to improve outcomes for patients undergoing IVF.

## Preimplantation genetic testing for aneuploidy or PGT-A

Assisted reproductive technologies (ART) have evolved into several categories and involve fertility treatments where the eggs or embryos are influenced, with the overarching objective being the birth of a healthy child for couples struggling to conceive naturally [13]. The most common ART procedure is in vitro fertilisation (IVF), which involves oocytes being fertilised outside the body, after which the resulting embryo is implanted into the uterus. In addition to IVF, preimplantation genetic testing (PGT) was developed to determine which embryo would be suited for implantation and produce a healthy pregnancy [13]. This testing involves the screening of embryos for a variety of genetic disorders to select the genetically healthy embryos to avoid the risk of implantation failure, miscarriage or a child with a genetic disorder. Therefore, allowing implantation success and pregnancy success is independent of factors that affect fertility, such as advanced maternal age, and also limits the transfer of multiple embryos to avoid multiple gestations [14]. A significant form of PGT that has emerged is preimplantation genetic testing for aneuploidy (PGT-A) [13]. This specific subset of PGT only focuses on detecting aneuploidy and chromosome abnormalities. Throughout its evolution, PGT-A has become more sensitive, lower in cost and has a faster turnaround time [15].

**Fig. 2:**
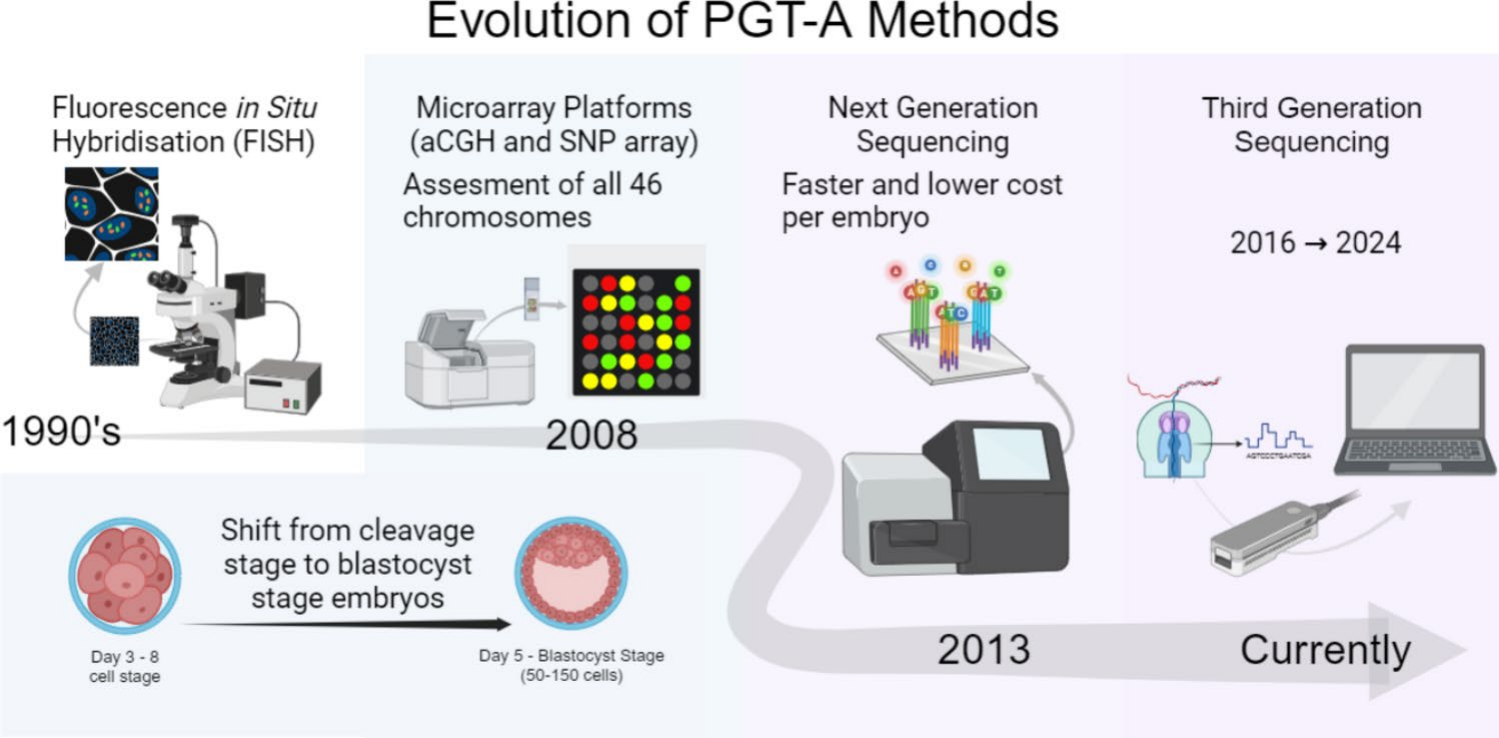
The changes in methods used for preimplantation genetic testing, starting with FISH using day 3 embryos, then to microarray methods, which shifted to using biopsies of day 5 cleavage stage embryos, to more recently the use of next-generation sequencing, which allows for faster results and lower cost per embryo. Currently, third-generation sequencing is being developed to improve upon previous methods. Adapted from “Microarray Editable Results (Layout)” by BioRender.com (2024). Retrieved from https://app.biorender.com/biorender-templates

Along with this evolution of technologies used for testing has come changing outcomes, with early research showing no benefits. Recent studies have demonstrated some benefits for PGT-A, where it was shown to reduce pregnancy loss and the number of embryo transfers required to achieve a similar number of live births compared to non-PGT-A groups. Despite these advances, other studies have found that PGT-A did not improve the live birth rate per patient [11, 16, 17]. As the practice of PGT-A has evolved, two key changes have been observed. The first change was the shift from extracting DNA from day 3 blastomeres to taking trophectoderm (TE) cells from the day 5 embryos (Fig. 2) [15]. PGT-A can still be conducted with polar bodies from oocytes and blastomeres; the limitation of blastomeres from day 3 cleavage stage embryos was the higher levels of mosaicism that did not represent the whole embryo. This results in a higher false positive rate than the day 5 embryos [18]. Therefore, day 3 cleavage stage embryos were considered less suited for implantation [18]. Further, TE cells allow 5–10 cells to be extracted compared to a single cell and thus, reduce the risk of false positives [18].

The second shift was the evolution of the testing practices (Fig. 2). Table 1 demonstrates some key differences between current PGT-A techniques and their capabilities. Preimplantation genetic diagnosis was the first iteration of chromosome testing to detect X-linked diseases through PCR. Later, with the addition of fluorescence in situ hybridisation (FISH) as a form of screening, it became possible to detect specific aneuploidies and structural abnormalities. However, this was limited to only screening a small number of chromosomes per test [18]. Since then, PGT-A has evolved to enable the screening of all pairs of chromosomes through various methods, including microarray platforms and next-generation sequencing technologies (Fig. 2) [18, 19].

**Table 1:** Comparison of methods commonly used for PGT-A, including microarray (aCGH and SNP array) and NGS. Including detection of chromosomal abnormalities, resolution, time, costs, and the embryo stage most commonly assessed with each method [20–26]

Methods	aCGH	SNP Array	NGS
Chromosomal abnormalities	Aneuploidy, mosaicism (40–60%)	Aneuploidy, mosaicism (50%), polyploidy	Aneuploidy, mosaicism (20–80%), polyploidy
Resolution	6–10Mb	6 Mb	1–3 Mb
Time	9–30 h	24 h	15–24 h
Costs	Approx. $5000 USD		$41 USD to $502 USD
The embryo stage most common with each method	Inner cell mass cleavage stage (day 3) and Trophectoderm biopsy of blastocysts (day 5)	Inner cell mass cleavage stage (day 3) and Trophectoderm biopsy of blastocysts (day 5)	Trophectoderm biopsy of blastocysts (day 5)

## Early PGT-A using fluorescence in situ hybridisation

As ART evolved, there came the hope of improving outcomes for patients with advanced maternal age, recurrent implantation failure and spontaneous abortions. The goal was to select chromosomally normal embryos to increase the pregnancy rate, leading to healthy children. Fluorescence in situ hybridisation (FISH) was the first method to assess for chromosomal abnormalities. As the name suggests, this method utilised chromosome-specific fluorochromes that would hybridise to chromosomes and, when looked at under a microscope, would emit a colour unique to the chromosomes assessed, thus allowing for the number of chromosomes to be determined [27]. Using this method, initially only 3–5 chromosomes could be evaluated at a time, and there was usually a variation of chromosomes 13, 15, 16, 18, 21, 22, X and Y. It is important to note that there are FISH methods capable of detecting all 24 chromosome pairs, referred to as spectral karyotyping (SKY-FISH) and multicolour FISH (M-FISH). Large-scale FISH is commonly used with other methods that provide direct visualisation of all chromosomes rather than for diagnostic purposes [28]. However, clinicians have not widely implemented this method in routine PGT-A due to the cost and complexity. In routine practice, cells were extracted from day 3 cleavage stage embryos, which, as discussed previously, had limitations due to only single cell or polar bodies being available for investigation. FISH became a popular method for chromosome assessment in the 1990 s and was first used to detect X-linked diseases [29, 30].

### Studies of FISH and outcomes for women with AMA

Many early studies sought to establish the benefits of FISH as a method for PGT-A for women of advanced maternal age and with recurrent implantation failure. However, several key limitations determined that FISH was not an apt method for PGT-A; the main issue being that there was no improvement in pregnancy and implantation rates between patients who underwent FISH testing compared to those who did not [30, 31]. It was further concluded that more chromosomes needed to be assessed during PGT-A [30, 31]. To overcome these limitations, two rounds of FISH analysis were conducted, which allowed for typically 6–7 different chromosomes to be assessed (chromosomes 15, 16, 22) [27, 32]. Though there was no significant improvement in implantation and ongoing pregnancy rates, the use of FISH did allow for fewer embryo transfers, which reduced the risk of multiple gestations [27]. When assessing if FISH for PGT-A could improve spontaneous abortion rates for women 35 years and older, Munne et al. looked at nine chromosomes to determine if the embryos were suitable for implantation (2006). These chromosomes included 13, 15, 16, 18, 21, 22, X, Y and either 1 or 17 using two rounds of FISH [33]. From 100 centres, 2279 cycles were included, with the average maternal age being 39.6 years. It was concluded that preimplantation genetic testing prevented 30% of spontaneous abortions, and in younger patients, there was a 27% reduction in pregnancy losses [34].

Two studies investigated PGT-A FISH results to determine correlations between advanced maternal age (women 35 years of age and over) and aneuploidy rate. Two studies investigated PGT-A FISH results and found that aneuploidy rates almost doubled between the ages of 35 and 44, from 54.5% (38 years of age) to 90% (44 years of age) and from 20% (35 years of age) to 40% (40 years of age) when assessing 1375 embryos and 20,000 oocytes, respectively [32, 35]. Despite some differences being investigated, these results indicated a clear trend of aneuploidy detection in advanced maternal age patients [32, 35]. Despite this correlation, several RCTs conducted on patients with advanced maternal age and recurrent implantation failure determined that FISH would not be suitable for PGT-A [36–38]. This conclusion was determined as each study screened 4–6 autosomal chromosomes as well as the sex chromosomes and failed to demonstrate any benefits of PGT-A regarding implantation rates and pregnancy rates [36–38]. However, Schoolcraft et al. observed an 11% increase in the delivery rate when using FISH [36–38].

FISH provided visualisation of chromosomes and was an early method used to establish a basis for PGT-A for patients undergoing IVF. Studies failed to find that FISH was suitable for application in PGT-A due to its inability to improve patient outcomes. A key reason was that FISH cannot screen less than half of all chromosomes and cannot detect mosaicism except on the specific chromosomes being tested [30, 35]. Further, standard FISH is no longer recommended for PGT-A as more comprehensive methods have been developed and FISH failed to provide significant clinical outcomes for patients undergoing PGT-A. Subsequently, microarray methods replaced FISH, as microarrays could screen all 23 chromosome pairs [39].

## Microarray platforms

The next step in advancing PGT-A was to use microarray technologies, of which two key types have been utilised for preimplantation genetic testing: array comparative genomic hybridisation (aCGH) and single-nucleotide polymorphism array (SNP array). Both microarrays use oligonucleotide probes that span the whole genome and range from 25 to 70 bp in length; these oligonucleotides hybridise to the surface of a glass slide with the sample being tested [1, 25, 40]. The key difference in these technologies is how samples are analysed; aCGH relies on analysing the ratio of oligonucleotide signals between patient samples compared to a normal control, while the SNP array utilised different signal colours from the SNPs identified at each oligonucleotide with analysis conducted on the B-allelic frequency of these SNPs without the need for normal controls included in the assay [41]. Though both methods are applicable for various forms of PGT, there are advantages and disadvantages for aCGH and SNP arrays due to the probe length, number of regions and spacing. Specifically, neither array can detect balanced translocations, inversions and insertions where there is no gain or loss of genetic material [42]. While aCGH can detect mosaicism at 10–20% within a population of cells, it cannot detect polyploidy or uniparental disomy (UPD), while an SNP array can detect both [25]. Another advantage of microarray for PGT-A was the ability to be used with whole genome amplification [43, 44]. However, whole genome amplification (WGA) has limitations that affect its reliability for PGT-A, including amplification bias, preferential allelic amplification and allelic dropout [19, 45].

### Applicability of microarray methods for PGT-A

Several studies were also conducted to determine the accuracy of PGT-A microarrays and whether these methods improved PGT-A outcomes, whereas FISH did not. Analyses of 52 blastocysts donated by 20 couples used aCGH to determine the ability to detect aneuploidy and mosaicism [18]. It was observed that 30% of the trophectoderm samples were euploid and 32.4% were mosaic and were able to obtain results for 100% of the samples through aCGH [18]. Microarray methods also examined differences between cleavage stage and blastocyst stage embryos. Several studies observed diminished aneuploidy rates from cleavage (day 3) compared to blastocyst (day 5). One study found a 70.6% aneuploidy rate and a 39.6% implantation rate at the cleavage stage compared to a 47.8% aneuploidy rate and a 49.2% implantation rate at the blastocyst stage across various age groups from 35 to 42 years of age [46]. A larger study looked at differences between 2204 embryos at multiple stages of development. Split into the corresponding groups: oocytes, cleavage stage and blastocyst. There was a significant decrease in the number of aneuploid errors detected in 58% of blastocysts compared to 75% of oocytes [10]. This trend resulted in the use of blastocysts for PGT-A becoming increasingly popular.

Patient outcomes in one study for patients with advanced maternal age found that aCGH improved live birth outcomes from 32.8% for non-PGT-A to 54.1% for PGT-A in only one group (Table 2) [47]. Other studies determined no or only a slight increase when using PGT-A for patients of advanced maternal age (AMA) [44, 48]. Despite these differences, it was consistently found that PGT-A allowed for fewer embryo transfers per patient, fewer embryo cryopreservations and fewer miscarriages in the PGT-A group [44, 48]. For patients with recurrent implantation failure (RIF) and recurrent miscarriages (RM), across two studies, there was an increase in live birth rates from 19–32.8% for non-PGT-A groups to 47.8–51.6% for RIF and 50–55% for RM for patients who underwent PGT-A [44, 47]. Lee et al. also included in their study that through PGT-A, there was an increase in live birth rates for patients using PGT-A that was close to oocyte donors, which had a live birth rate of 57.1% [47]. Whilst there was a statistically significant increase, it did caution that many fertility-based problems, especially for patients with advanced maternal age, recurrent miscarriage and implantation failure, are complex and can be owed to several factors beyond simply the patient’s age. Therefore, it was recommended that due to current limitations around the cost of PGT-A via aCGH, it should be offered in specific cases and not for all patients undergoing IVF procedures [44].

**Table 2:** Comparison of several studies that compare the use of microarrays in PGT-A for patients with advanced maternal age (AMA), recurrent implantation failure (RIF) and recurrent miscarriage (RM) [44, 47, 48]

Authors	No PGT-A			With PGT-A		
	Live birth rates (AMA)	Live birth rates (RIF)	Live birth rates (RM)	Live birth rates (AMA)	Live birth rates (RIF)	Live birth rates (RM)
[47]	32.8% (20 of 61)			54.1% (33 of 61)	51.6% (33 of 64)	55.9% (38 of 68)
[44]	24.3% (48 of 197)	19% (8 of 42)	12.5% (5 of 40)	35.2% (18 of 51)	47.8% (11 of 23)	50% (9 of 18)
[48]	24% (45 of 191)			24% (50 of 205)		

While there were some discordant results related to the benefits of aCGH microarrays PGT-A, it was found to improve diagnostic accuracy from 0.5 to 16% [49]. Despite its improvements, several limitations have remained associated with aCGH and other microarrays beyond the detection of polyploidy and mosaicism when they are not present in a large population of cells. In particular, there are a large number of probe designs that have low specificity, as well as millions of SNPs that are unrepresented [1, 40, 42]. While microarrays have been demonstrated to be reliable and accurate when detecting aneuploidies, errors still occur. A further limitation of aCGH is the high cost associated. In 2020, Neumann et al. found that PGT-A cost when using aCGH on polar bodies was approximately $5173 USD not including the cost of IVF, which was found to range from $15,000 USD to $2000 USD depending on which countries PGT-A and IVF were being conducted in Neumann et al. [23]. However, an SNP array is typically more cost-effective and a popular method of PGT-A as a result [50]. While microarray methods significantly improved PGT-A outcomes, the limitations, specifically those associated with cost and mosaicism detection, have led to next-generation sequencing platforms becoming the next phase of PGT-A [49, 51].

## Next generation sequencing

In recent years, next-generation sequencing (NGS) has overtaken FISH and microarray assays as the next step in the evolution of PGT-A. Though most platforms work by using direct reads from genomic sequencing fragments, these reads are short in length, typically 100–300 bp, and are quantified according to sequence read number [39]. NGS also expanded the multiplex potential, allowing multiple samples to be sequenced, making them more cost-effective at approximately $100 USD per sample [52]. NGS platforms also work with WGA, which is beneficial when only a small number of cells can be extracted from an embryo as well; NGS has been shown to accurately detect aneuploidy with only a small amount of genome 0.1 × to 1 × coverage depth compared to microarray [52, 53]. When comparing NGS to SNP array results from analysing samples of 105 blastocysts, it was found that NGS was able to detect mosaicism at lower fractions than SNP arrays. However, the authors noted that results could be affected if noise or amplification bias were present in the samples [54]. Several studies were also able to demonstrate that NGS platforms were faster than microarray methods, particularly through the addition of WGA as an initial amplification method, reducing the time of sequencing down to 16–24 h [6, 26, 45]. Along with improvements in sequencing technologies, there have also been improvements in methods of DNA amplification. A key advantage of such methods is the ability to amplify from a minimum amount of DNA as low as 10 pg with high genome coverage. However, the risk of allelic dropout can pose a risk when using WGA. Thus, several variations of WGA exist, which differ in the enzyme used and the number of priming events [55]. However, various WGA methods may not always apply to PGT-A sequencing methods. Two common WGA techniques are multiple displacement amplification (MDA) and multiple annealing and looping-based amplification cycles (MALBAC). Illumina have also developed a method of whole genome amplification known as Sureplex, which is commonly used with the Veriseq PGS kit. Sureplex amplifies DNA through semi-random, non-self-complementary primers to produce 2–5 µg of DNA from either a single cell or embryo biopsies. Sureplex was compared to MALBAC was found to be better suited for copy number analysis as MALBAC had higher non-uniformity across the genome which can increase the risk of false positives for copy number variants [56].

While WGA used in conjunction with NGS has been shown to be effective, other methods have been continually developed. Studies have also published methods of PGT-A that utilised Targeted NGS. Targeted NGS varies from standard NGS through the amplification of specific regions through the use of custom primers that amplify a specific point of interest within the human genome. In 2012, a technique known as the Fast Aneuploidy Screening Test Sequencing System (FASTSeqS) was developed by Kinde et al. for non-invasive prenatal screening with NGS [57]. This method aimed to simplify the process of DNA amplification through a primer pair that would amplify SNP regions, specifically Long Interspersed Nuclear Element (LINE-1) repeats, which comprise 17% of human DNA and are found on all 23 chromosome pairs [57]. In 2022, this method was assessed for PGT-A using 190,000 samples from trophectoderm biopsies, where results identified aneuploidy in 47.2% of samples, as well as other chromosomal abnormalities such as polyploidy and UPD with 100% sensitivity and specificity [57, 58]. An alternate targeted NGS method study used the Ion Torrent by ThermoFisher and a total of 77 biopsies from blinded biopsies of aneuploid and euploid samples originating from 31 embryos against 24 chromosome qPCR. Samples were concordant in 98.7% of cases and found a false positive rate of 0.23% in embryos at the chromosome level [59]. Another used a method known as PGTSeq-A using the NextSeq 500/550 (Illumina) to carry out a blinded, non-selection study. All embryos were transferred on the basis of morphology [60]. Disclosure of results was unblinded after 13 weeks’ gestation, so diagnostic prenatal screening could be implemented if the patient chose to do so. Of the 402 study participants, there were 484 single frozen blastocyst transfers which results in 2110 blastocysts being analysed. It was found that 0.6% failed to amplify, 2.2% non-concordant result, no call rate of 2.8%, 60.2% classified as euploid and 24.5% were aneuploid whole chromosome; secondary findings also reported 3.5% as aneuploid mosaic and 8.8% as segmental aneuploidy [60]. Of the 312 embryos that were classified after implantation, 82.1% (256/312) were euploid and 64.7% (202/312) reached ongoing pregnancy and healthy gestations. Embryos that were found to have been aneuploid after implantation 0% progressed to an ongoing pregnancy or clinical pregnancy. There was a low call error rate determined to be 0 to 2.43%. It should be noted that for this study, half of the participants were under 35 years of age, which is usually a key focus for PGT-A-based studies due to the age-related nature of aneuploidy. The authors state that this was done to focus on the embryo roles in infertility and excluded patients with poor reproductive histories, in line with other studies [60]. So, whilst whole genome amplification methods are commonly used, targeted NGS methods also provide accurate PGT-A outcomes.

**Fig. 3:**
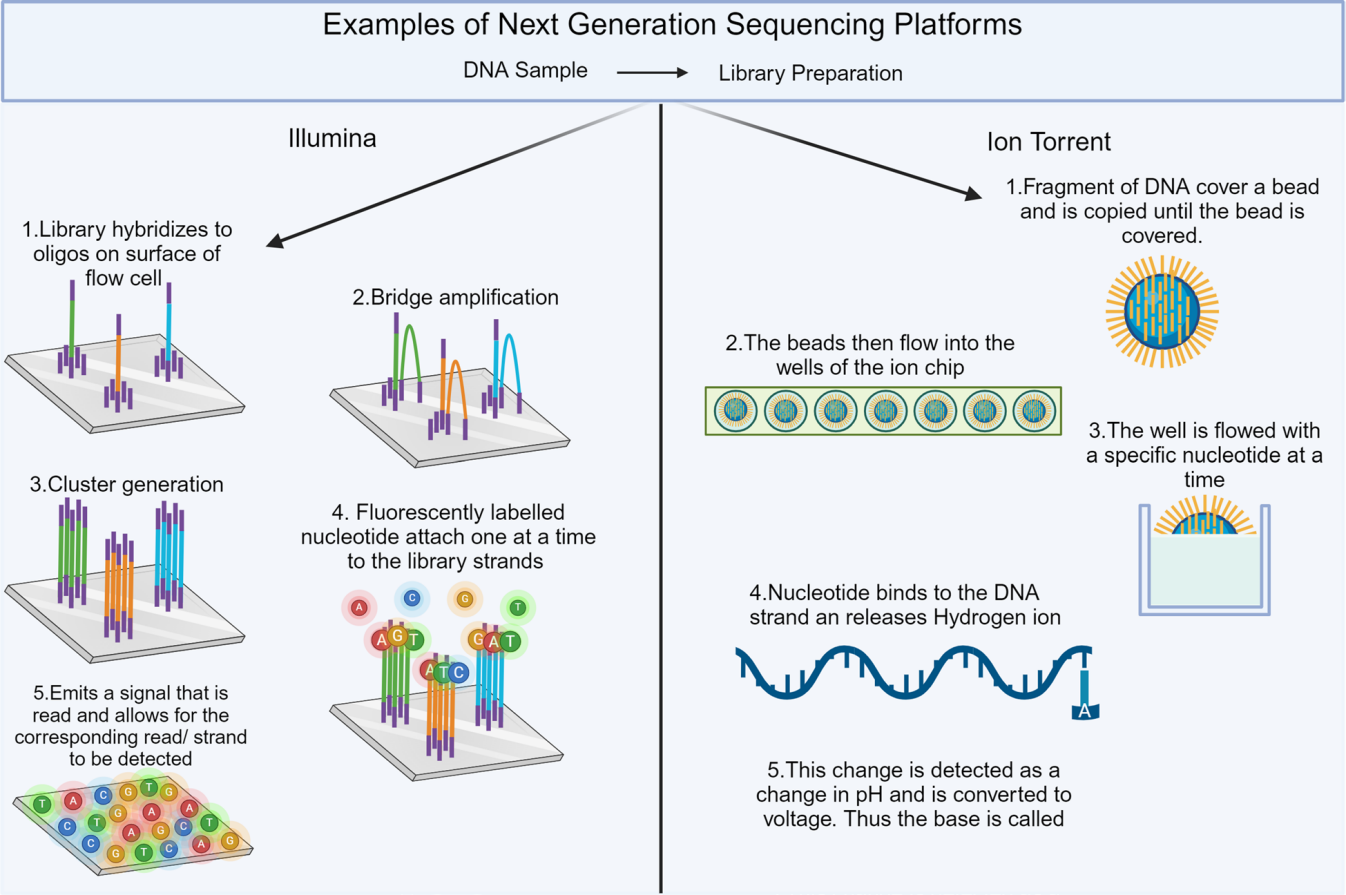
The process of DNA sequencing through either fluorescently tagged probes and hydrogen ions utilised in next-generation sequencing of two common platforms Illumina and Ion torrent. Adapted from “Next Generation Illumina”, by BioRender.com (2024). Retrieved from https://app.biorender.com/biorender-templates

Illumina and ThermoFisher have developed two of the most commonly utilised PGT-A platforms, the MiSeq and Ion Torrent PGM platforms. Each platform has a compatible aneuploidy sequencing method. For PGT-A, the VeriSeq PGS Kit can be used with the MiSeq platform, which can obtain results for 24 samples in 12 h per run [20]. VeriSeq can be used for whole chromosome aneuploidy and mosaicism detection at lower percentages from 20 to 80% [61–63]. However, it was not initially designed for the automatic detection of structural abnormalities. One study found it could detect structural abnormalities as small as 5 Mb [61, 62]. As previously stated, the MiSeq sequencer is commonly used with the VeriSeq Kit, Illumina NGS sequencers use sequencing by synthesis, where DNA fragments hybridise oligos on the surface of a flow cell (Fig. 3). The fragments then undergo clonal amplification and form clusters on the surface of the flow cell. The addition of fluorescently labelled nucleotides releases a fluorescent signal corresponding to the specific nucleotide, allowing for the nucleotide to be called.

ThermoFisher has its own PGT-A method known as the Ion ReproSeq Kit, which, depending on which version of the kit, is compatible with various ThermoFisher NGS platforms, including the Ion Torrent. This kit includes all necessary components from single-cell or multiple-cell DNA amplification to the multiplexing library preparation, allowing up to 96 samples to be sequenced in a single assay and taking under 11 h to sequence 48 samples. IonRepro Seq can also detect structural abnormalities as well as mosaicism at 30% and segmental aneuploidies [61]. Ion Torrent uses a different method of sequencing to Illumina, once the library steps have been completed it is loaded onto a semiconductor chip, which contains wells. Beads present in the keep will bind single strand of DNA to it and be replicated until the entire beads are covered at which stage each bead will flow into its own well (Fig. 3). The chip is flooded each nucleotide at a time, as each nucleotide is incorporated it releases a hydrogen ion, which changes the pH of the solution which is detected as a change in voltage and allowing for the signal to be called.

While both methods offer different ways of carrying out PGT-A, both provide similar high-throughput, accurate aneuploidy and mosaicism detection at rates significantly lower than previous microarray methods. Table 3 summarises some similarities and differences between the two NGS methods. An early 2014 study looked at the two Ion Torrent and MiSeq platforms [53]. This study used cells from 18 samples of spontaneous abortion to determine the performance of each platform on the GC bias level and aneuploidy detection. It was concluded that there was no statistically significant difference between the two platforms; however, it was noted that a larger sample size would be needed to investigate their clinical utility more thoroughly [53]. More recent studies further found that there was high concordance between the two platforms when comparing the automatic (Ion S5) and manual (MiSeq) identification of PGT-A [64]. Of 107 clinical biopsies, 3 of the embryo samples could not be obtained results due to “chaotic patterns”, as such, ploidy could not be determined. The results were concordant for 103 biopsies, and only one result was non-concordant; one embryo was identified as euploid on the Ion 5S and aneuploid or mosaic on the MiSeq; the overall concordance was 99.04% per embryo [64]. Cell lines were mixed to generate varying percentages of mosaicism. For the cell lines, the specificity was 100%, and for sensitivity, it was 93% for the IonS5 and 90% for the MiSeq which was not a significant result [64]. Overall, it was found that the ThermoFisher Ion system was faster through the automatic identification and required less manipulation time compared to MiSeq. Though the automatic system was less flexible, it still provided a more standardised approach and faster time to results [64]. This was further seen in a similar study that compared VeriSeq with ReproSeq, looking at whole and segmental mosaicism. For whole mosaicism detection, cell lines were used to generate mosaic-like samples at varying percentages from 10 to 100%. For whole chromosome aneuploidy, false negatives were only observed with a mosaicism of 10% for VeriSeq and 20% for ReproSeq [61].

**Table 3:** Comparison of two NGS PGT-A methods, the VeriSeq developed by Illumina and the Ion ReproSeq developed by ThermoFisher [61, 64–66]

Methods and platform	VeriSeq (MiSeq)	Ion ReproSeq
**Manufacturer**	Illumina	ThermoFisher
**Compatible platform**	MiSeq	Ion S5 and Ion Torrent
**Run details**	24 samples per run in 14 h	96 samples per run 11 h to sequence 48 samples
**Read size**	150 bp or 400–550 bp 1 × 36bp–2 × 300 bp 36 bp are read	200 bp 100–150 bp on Ion S5
**Amplification**	Sureplex (WGA)	IonSingleSeq (WGA) included
**Mosaicism**	20–80%	30–80%
**Aneuploidy detection**	Separate WGA and library Manal calling Longer time to results	Incldued WGA and library in one kit Automatic calling Faster but less flexible

### Next generation sequencing and PGT-A outcomes

When comparing morphological assessment and PGT-A, there has been varying improvement of approximately 5–14% observed in ongoing pregnancy and live birth rates for patients who underwent PGT-A using NGS [17, 67]. When NGS was compared to aCGH, there was a significant increase in the ongoing pregnancy rate per transfer from 71% for aCGH to 85% for NGS (Table 1) [68]. More recently, 1974 blastocysts from patients with AMA were tested for PGT-A via NGS compared to 1576 blastocysts for non-PGT-A and found that NGS was able to lower miscarriage rates from 14.7% in the non-PGT-A group to 2.3% in the PGT-A group and increase the live birth rate from 30.9 to 52.1% [69]. This was also seen in over 18,000 embryo transfers with PGT-A and over 19,000 transfers with no PGT-A from 2017 to 2020; looking at live birth rates found an increase for women over 35 years old from 42.7 to 48.9% (Table 2)[70].

**Table 4:** Comparison of studies to compare outcomes for miscarriage and live birth rates using PGT-A through NGS to improve outcomes [67, 69, 70]

	Miscarriage rates		Ongoing pregnancy/live birth rate per transfer	
Authors	Non-PGT-A	PGT-A	Non-PGT-A	PGT-A
[67]^a^	9.6% (27 of 313)	9.9% (27 of 274)	50% (137 of 274)	45.7% (143 of 313)
[70]	21.2% (1402/12,372)^b,^^d^ 23.2% (931/7128)^c^	17.9% (995/9211)^b^ 17.9% (976/9014)^c^	41.7% (5166/12,372)^b^ 42.7% (3043/7128)^c^	48.7% (4483/9211)^b^ 48.9% (4410/9014)^c^
[69]	14.7% (90/612)	2.3% (13/568)	30.9% (189/612)	52.1% (296/568)

^a^Did not include live birth rate, but included ongoing pregnancy rate

^b^Single embryo fresh and frozen embryo transfer

^c^Only single embryo transfer

^d^Not statistically significant

There have also been studies that investigated the rates of patients with recurrent implantation failure (RIF) and recurrent pregnancy loss (RPL) when an NGS PGT-A test was used [71, 72]. Shi et al. observed improvements in the implantation rate (64.2%), the pregnancy rate (57.5%) and the live birth rate (45%), compared to the non-PGT-A group (38.3%, 33.3% and 28.4%, respectively) from their cohort of 212 RPL and 66 RIF patients [71]. However, these rates were not significantly higher within the RIF group than in the non-PGT-A group [71]. This finding was also observed by Tong et al., who observed no significant difference between the implantation rates and clinical pregnancy rates when PGT-A was used (2021). Therefore, whilst NGS is advantageous for AMA and RPL, there have not been significant increases regarding outcomes for patients with RIF, likely due to the complex nature of these conditions.

Whilst one key advantage of NGS platforms is the ability to detect mosaicism at lower percentages than microarray platforms, there are still several difficulties. It was found that, on average, in 2.5% of cases, no NGS results were obtained due to amplification failure or insufficient data [60, 73, 74]. In these studies, when blastocysts were biopsied, 3.5% were mosaic, and 8.8% were segmental, while in another, 44% of samples had either an aneuploid result or no result [60, 74]. More recent studies found similar results, with 8% of those biopsies showing mosaicism or segmental mosaicism for aneuploidy, while 54% of blastocysts were true aneuploidy [75]. NGS significantly changed patients’ outcomes for PGT-A through an improved understanding of mosaicism with the advent of trophectoderm biopsy in clinical practice and its ability to detect mosaicism when it is present in a smaller population of cells. However, there remain several limitations regarding the use of these platforms, including the risk of amplification bias during amplification processes, as well as allelic dropout and preferential allelic amplification [6, 45]. Whilst the cost per assay is low compared to previous PGT-A methods (between $41 USD and $502 USD), these platforms themselves are also costly and can range from $80,000 USD to $654,000 USD [24]. This means that some individual IVF clinics cannot house these platforms and therefore require that samples be sent to larger external laboratories for testing [6, 26, 45]. NGS platforms also yield large datasets, and these require complex bioinformatic analysis, which may increase the time before a result can be obtained, which, along with the need for embryos to undergo cryopreservation for transportation, can further increase the time taken before final results are disseminated [26, 76].

## Third generation sequencing

In recent years, a new form of DNA sequencing has emerged, dubbed “Third Generation Sequencing” or “Long Read Sequencing”, which can provide sequencing results in real-time. These methods developed by multiple platforms, including Oxford Nanopore Technologies (ONT), PacBio, Illumina and BGI (as of September 2024), may address some of the current limitations of PGT-A methods. Currently, for PGT-A, the only studies published that utilised third-generation sequencing are based on the ONT platforms [77].

### PGT-A testing using nanopore sequencing

This technology begins to sequence DNA through a Y-shaped adapter, which guides the DNA to the surface of the individual nanopore. A single strand is guided through the nanopore, which is 10 nm in diameter, by a motor protein attached through the library preparation steps [77, 78]. Nanopore sequencing has also been developed to be faster than NGS, with a maximum of 420 nucleotides sequenced per pore per second, making it 1200–10,000 times faster than traditional NGS platforms [78]. Another advantage of nanopore sequencing over NGS is the ability to complete real-time base-calling, allowing results to be obtained faster, whereas, for other NGS platforms, the base-calling step can only be completed once the sequencing is finished [78].

The first published results to determine the applicability of nanopore sequencing for PGT-A were in 2016 by Wei and Williams [78]. WGA was used on four DNA samples from a male with trisomy 12, a male with trisomy 21, a female with monosomy X and a karyotypically normal female and male; the amplified DNA was fragmented to less than 100 bp length strands. The samples were then sequenced, and 43,000 to 87,000 raw reads were obtained, of which 39.1–69.7% were mapped uniquely after 4 h of sequencing. All five samples correctly identified their karyotype following bioinformatic analysis [78]. This work was subsequently expanded to investigate trophectoderm cells from day 5 embryos and looked to optimise further the previous study [77]. A total of 9 samples were tested, and they found that within 2 h, enough data was obtained for aneuploidy detection, while within a further 1–4 h of sequencing time, mosaicisms and large CNV could be accurately detected [77]. This work found that sequence data from 31,779 to 52,040 unique reads per sample was obtained from a sequencing run that took approximately 5 h and 15 min [77]. This could mean that cells could be obtained, amplified and sequenced within the same day, limiting the need for cryopreservation.

Since these two early studies, more work has been carried out regarding the applicability of nanopore sequencing. Wei et al. took 218 samples from chorionic villus sampling, amniotic fluid samples and trophectoderm biopsies using short-read transpore rapid karyotyping (STORK) [79]. From the trophectoderm biopsy samples, nanopore was 98.1% concordant with other PGT-A results. They also identified that this test could be feasibly performed for between $50 USD and $200 USD, depending on the number of samples sequenced together [79]. More recent studies have also shown 98.86% concordance for aneuploidy cell lines, 98.89% concordance for segmental aneuploidy detection and 97.92% concordance for the trophectoderm biopsy across the MiSeq NGS platform and nanopore sequencing platform [80]. To further support this, other studies also found a high concordance rate between nanopore sequencing and aCGH when testing polar body samples while also highlighting the benefits of nanopore sequencing in which 98.7% of samples were able to complete the workflow in 5 h for one sample, or within 16 h for 12 samples [81].

As such, nanopore sequencing is a rapidly developing technology, and since 2016, this platform has demonstrated that it can be cost-efficient and time effective. With the determined cost per assay ranged from $100 USD to $150 USD which matches NGS platforms such as VeriSeq, which costs between $120 USD and $150 USD when multiple samples are sequenced in one run [77, 80]. These platforms also have a significantly lower capital cost when compared to NGS platforms, ranging from $80,000 USD upwards, whereas the MinION starter pack begins at $2000 USD and from $9500 USD for the MinION and PromethION (2024) [77, 80]. There is potential that IVF clinics could integrate nanopore platforms to perform PGT-A in-house and would not need external laboratories, reducing the time from cell extraction to implantation [77]. However, re-validation of nanopore-based assays performed in-house may be a barrier to the wider implementation of this technology.

## Discussion and future direction

As sequencing technologies have evolved, PGT-A has aimed to improve outcomes for patients undergoing IVF through the selection of embryos with the highest chance of implantation and healthy offspring. Specifically for patients with a higher likelihood of experiencing pregnancy loss due to the presence of aneuploidies within an embryo. As methods of screening embryos for PGT-A have evolved, outcomes for patients have improved, though not only increasing likelihood of implantation but also reducing risk of miscarriage and selection of a single of embryo reducing the risk of multiple gestations. Several studies have demonstrated the benefits of PGT-A in improving these outcomes for patients with advanced maternal age, recurrent implantation failure and recurrent miscarriage [47, 69, 70]. As sequencing methods of PGT-A have changed the cost per embryo has lowered significantly over early methods, combined with the costs of IVF, it remains an expensive procedure and may not be as effective for all patients undergoing IVF. The only benefits of PGT-A has been for patients with a higher risk of aneuploidy compared those without [82]. 

Though PGT-A has progress with the intent of increasing the benefit to patients, it is important to address the limitations of PGT-A. In the presence of multiple cells with different chromosome copy number, some cells may be euploid and others contain an aneuploidy. Thus, the detection of mosaicism still remains an important factor in embryo testing as the transfer of such embryos potentially increases the risk of miscarriage or non-viable offspring [39]. However, mosaicism may only represent one part of the embryo not the entire embryo. Mosaicism detected from cells from the TE biopsy even if multiple cells are extracted may not be representative of the inner cell mass chromosome makeup [39]. As NGS has evolved, it has improved the detection of mosaicism at lower percentages over previous methods. The transfer of such embryos carries the risk of miscarriage or non-viable offspring; however, the clinical significance of transferring mosaic embryos is still under investigation; however, some studies shave suggested that mosaic embryos have the ability to self-correct and can still produce a healthy embryo. For couples with few embryos, this may provide an option and help to prioritise which embryos the select [39, 83, 84].

Another limitation of PGT-A is the potential for harm the embryo through biopsy, which may affect implantation or PGT-A results and give an unreliable result; it is recommended that multiple cell biopsy reduce the risk of damage affecting the result [85, 85],Group et al., 2020). As results of this, several studies have looked at the possibility of using cell free DNA obtained from the media from which embryos are cultured in. However, this requires more investigation and may become a tool to prioritise certain embryos than definitively selecting an embryo for implantation [85–87].

In future, the continued development of new methods and technologies through companies such as ONT, PacBio and Illumina may address the current limitations of PGT-A. Though these methods are still in the research stage, the continued improvement in accuracy may eventually lead to their applicability for clinical use, especially with optimised library protocols, which would reduce time to sequencing and improve DNA quantity and quality. There is also the potential for machine learning and artificial intelligence to aid in the process of PGT-A. Machine learning methods are already being developed to predict embryos at risk of aneuploidy or mosaicism, which may benefit from PGT-A screening [88]. Continuing advancements may offer several solutions to the current PGT-A limitations.

## Conclusion

Since the early PGT-A techniques, outcomes for patients with AMA, RIF and RM have improved significantly. The process of obtaining results from PGT-A can take 7–21 days; however, more recent technologies such as nanopore sequencing may be able to reduce these times [78]. Furthermore, advances in sequencing techniques have improved current PGT-A and provided greater accessibility to IVF clinics, circumventing the need to outsource. Further improvements in sequencing for PGT-A methods will not only improve outcomes of PGT-A but also reduce the cost and stress couples face through the IVF process. This field will continue to advance with technological advancements and the development of new long-read sequencing methods through increasing competitors.

## Data Availability

No datasets were generated or analysed to produce this review.
